# ReAlign-P: a vertical iterative realignment method for protein multiple sequence alignment

**DOI:** 10.1093/bioinformatics/btaf421

**Published:** 2025-07-25

**Authors:** Yixiao Zhai, Pinglu Zhang, Quan Zou, Ximei Luo

**Affiliations:** Institute of Fundamental and Frontier Sciences, University of Electronic Science and Technology of China, Chengdu, 610054, China; Yangtze Delta Region Institute (Quzhou), University of Electronic Science and Technology of China, Quzhou, 324003, China; Institute of Fundamental and Frontier Sciences, University of Electronic Science and Technology of China, Chengdu, 610054, China; Yangtze Delta Region Institute (Quzhou), University of Electronic Science and Technology of China, Quzhou, 324003, China; Institute of Fundamental and Frontier Sciences, University of Electronic Science and Technology of China, Chengdu, 610054, China; Yangtze Delta Region Institute (Quzhou), University of Electronic Science and Technology of China, Quzhou, 324003, China; Institute of Fundamental and Frontier Sciences, University of Electronic Science and Technology of China, Chengdu, 610054, China

## Abstract

**Motivation:**

Reliable protein multiple sequence alignment (MSA) is essential for downstream biomedical research and directly impacts the accuracy of analytical results. However, protein sequences often exhibit low similarity and complex alignment patterns, and existing general alignment tools frequently fall short in terms of accuracy. Many current realignment methods are outdated, suffering from issues such as code obsolescence and inadequate precision. As a result, there is a pressing need for realignment methods that can better address these challenges.

**Results:**

This study introduces ReAlign-P, a realignment tool designed specifically for protein MSA. ReAlign-P first divides the initial alignment into three regions and applies a novel vertical iterative realignment strategy to optimize the more conserved middle region. This method is by default compatible with MUSCLE5 for realignment, leading to a significant improvement in accuracy. We evaluated initial alignments generated using 10 different MSA parameter configurations across four protein benchmark datasets. The results demonstrate that ReAlign-P consistently outperforms or matches the quality of the initial alignments in all cases. In contrast, RASCAL—the only other currently functional protein realignment tool—sometimes even reduces alignment quality. ReAlign-P not only delivers more substantial improvements but also exhibits greater stability, effectively addressing the gap in available protein realignment tools.

**Availability and implementation:**

The source code and test data for ReAlign-P are available on GitHub (https://github.com/malabz/ReAlign-P).

## 1 Introduction

Protein multiple sequence alignment (MSA) is a cornerstone of biomedical research and underpins a wide range of downstream analyses. Its accuracy has a cascading impact on critical applications such as protein structure prediction, functional annotation, phylogenetic reconstruction, and drug target discovery (Wang *et al.* 2024). However, the high diversity and low similarity of protein sequences pose significant challenges for commonly used progressive alignment methods (Zhou *et al.* 2024). These methods typically follow the “once a gap, always a gap” strategy—once a gap placement error occurs in the early stages of alignment, it tends to propagate through subsequent steps, leading to systematic biases that are difficult to correct (Chao *et al.* 2022). To address this limitation, current strategies typically adopt two complementary solutions: (i) incorporating iterative refinement techniques during the alignment construction phase to repeatedly adjust and optimize the alignment, helping escape local optima and improve overall accuracy; and (ii) employing realignment methods in the post-processing stage to locally correct and refine the initial alignment, thereby enhancing quality without the need to rebuild it from scratch.

Iterative refinement is a common MSA optimization strategy that splits an initial alignment into two subsets, realigns them, and retains the version with a higher objective score. This process, using sequence-to-profile or profile-to-profile alignment, repeats until convergence or a set iteration limit is reached. Based on the partitioning strategy, existing iterative refinement methods can be broadly categorized into two types. The first is horizontal partitioning, which divides sequences directly. This is the most commonly used approach in mainstream tools and includes three main forms: (i) Random partitioning, used in tools like ProbCons (Do *et al.* 2005) and MSAProbs (Liu *et al.* 2010), where sequences are randomly split into two subsets to realigned; (ii) Single-sequence partitioning, adopted by tools such as MLAGAN (Brudno *et al.* 2003) and MANGO (Zhang *et al.* 2007), which divide a single sequence and the remaining sequences into two subsets for comparison; and (iii) Tree-based partitioning, implemented in tools like MUSCLE (Edgar 2004) and MAFFT (Katoh and Standley 2013), where sequences are grouped based on the topology of a guide tree. The second type is vertical partitioning, which divides sequences based on column-level information from the alignment. For example, FAMSA (Deorowicz *et al.* 2016) and QuickProbs2 (Gudyś and Deorowicz 2017) identify columns containing gaps, group sequences with gaps into one subset, and those without gaps into another, and then realign the two groups. Although this still results in two subsets, the partitioning logic is fundamentally different from horizontal strategies.

However, the iterative refinement mechanisms built into MSA construction have limited capacity to further improve alignment quality. To address this, researchers in recent years have developed various post-processing methods that operate independently of the alignment construction process. These tools function as standalone modules, aiming to refine and enhance existing alignments. Based on their partitioning strategies, they can be broadly classified into three categories: horizontal partitioning, vertical partitioning, and a combination of both. Horizontal partitioning realigners follow a similar approach to horizontal iterative refinement, dividing sequences into subsets and realigning them iteratively. Representative tools in this category include ReAligner (Anson and Myers 1997), the Remove First (Wallace and Higgins 2005), REFINER (Chakrabarti *et al.* 2006), and ReformAlign (Lyras and Metzler 2014). These adopt iterative optimization strategies to gradually enhance alignment quality. TreeRefiner (Manohar and Batzoglou 2005), although also based on horizontal partitioning, differs in that it employs 3D dynamic programming for re-alignment rather than relying on traditional iterative sequence strategies. In contrast, vertical partitioning focuses on the column-wise structure of the alignment. These methods segment the alignment into local regions or “blocks” and perform localized re-alignment to improve quality. Typical tools include Refin-Align (Mokaddem *et al.* 2019), SpliVert (Zhan *et al.* 2020), and RPfam (Wei *et al.* 2022), which enhance overall alignment accuracy by correcting specific low-quality regions. Other methods, such as RASCAL (Julie D. Thompson *et al.* 2003) and our previously developed ReAlign-N (Zhai *et al.* 2024a), adopt a hybrid strategy that combines both horizontal and vertical partitioning. For example, RASCAL first clusters sequences into subfamilies using Secator (Wicker *et al.* 2001), then identifies global and local core blocks based on the NorMD objective function, and finally realigns the detected low-quality regions. ReAlign-N takes a similar approach, first performing a global horizontal realignment of all sequences, followed by vertical segmentation and fine-tuning of identified low-quality regions, significantly boosting overall alignment quality.

While previous methods have improved alignment quality to some extent, sequence-based iterative refinement and horizontal partitioning often retain initial misaligned gaps, reducing overall effectiveness. Column-based refinement can better correct local gap errors but suffers from instability due to random partitioning. Moreover, in our tests, only RASCAL functioned reliably among existing post-processing tools, as most others were unusable due to broken links or inaccessible code.

In this study, we propose ReAlign-P, a post-processing realignment method for protein MSA. Drawing inspiration from SpliVert, ReAlign-P first segments the initial alignment into three regions—head, middle, and tail—along the sequence direction from the N-terminus to the C-terminus. Building on this framework, it introduces an innovative vertical iterative realignment strategy targeting the middle region to enhance the conserved region. To thoroughly assess the effectiveness of ReAlign-P, we conducted a systematic empirical study using four authoritative protein benchmark datasets. The experiments were conducted using initial alignments generated by 10 configurations of 6 mainstream MSA tools, including both versions with and without internal iterative refinement. The results show that, regardless of whether internal refinement was applied to the initial alignment, ReAlign-P consistently maintained or improved alignment quality across all datasets and effectively reduced the performance gap between different configurations. Moreover, in terms of both accuracy and stability, it outperforms RASCAL, currently the only publicly available post-processing tool for protein MSA.

## 2 Methods

### 2.1 ReAlign-P overview

The input of ReAlign-P is an initial alignment generated by an MSA tool from a set of unaligned protein sequences. Since MSA tools do not insert gaps evenly across sequences, the middle region usually has fewer gaps than the ends. This is because the core part of a protein sequence tends to be more conserved, while the terminal regions are more prone to insertions and deletions (indels). ReAlign-P first identifies regions requiring realignment. Inspired by the SpliVert tool, ReAlign-P focuses optimization on the central, more conserved regions of the alignment, while excluding the terminal regions, which are often subject to natural variation or structural disorder and thus prone to introducing errors if realigned indiscriminately.

**Figure 1.**
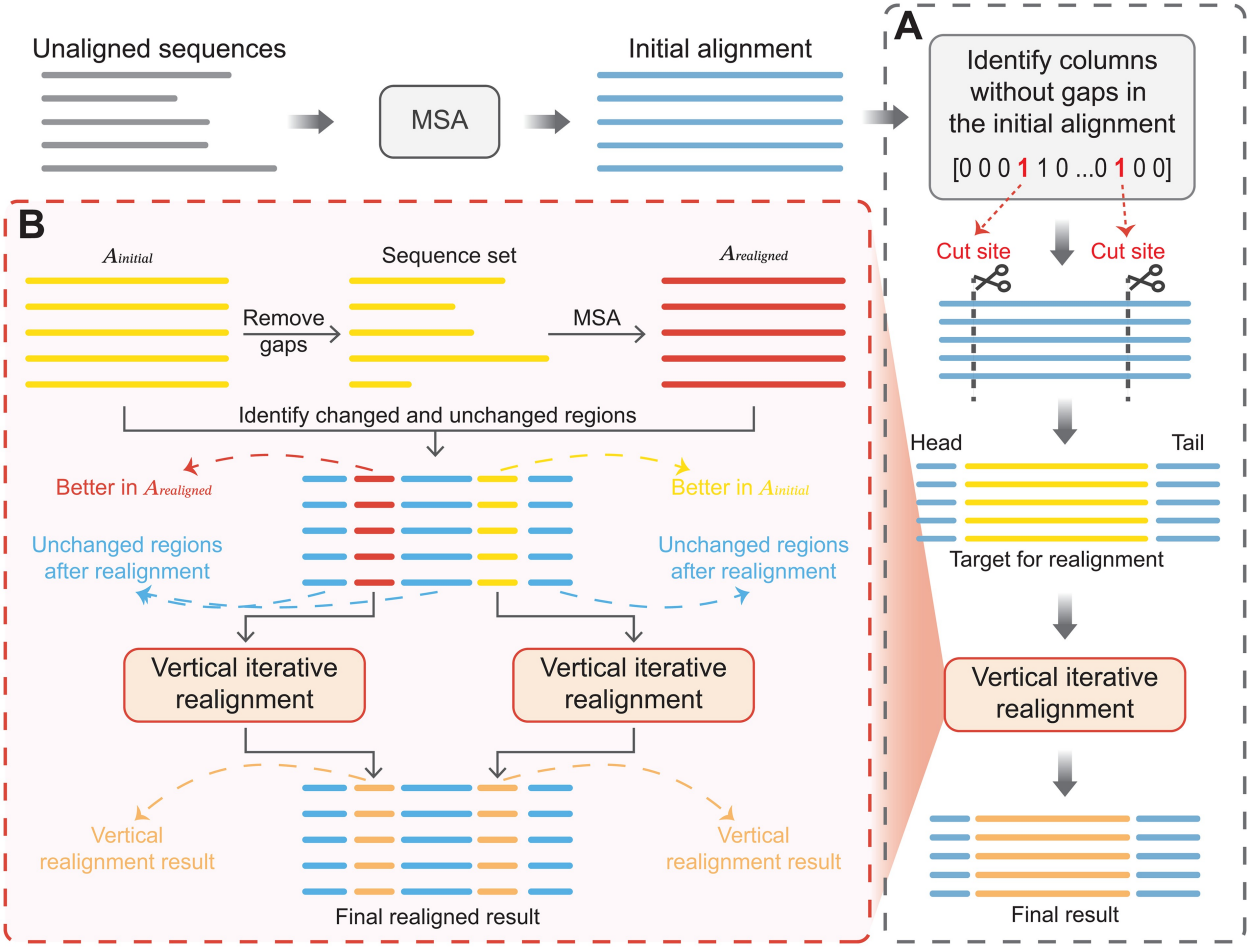
Schematic illustration of the ReAlign-P workflow and the vertical iterative realignment process. (A). The workflow of ReAlign-P. Starting from the initial alignment, ReAlign-P first identifies the first and last columns without gaps and uses them as cut sites to divide the alignment into three regions: the head, the target region for realignment, and the tail. The middle section (target region for realignment) undergoes vertical iterative realignment and is then merged with the head and tail regions to produce the final result. (B). The flowchart of vertical iterative realignment. The input for this stage is the region to be realigned, referred to as $A_{\mathrm{initial}}$. First, all gaps are removed to create a sequence set. This set is then realigned using the selected MSA tool to generate $A_{\mathrm{realigned}}$. Next, $A_{\mathrm{initial}}$ is compared with $A_{\mathrm{realigned}}$ to identify the unchanged regions and the changed regions in the alignment. The unchanged regions are left untouched, while the changed regions are evaluated using the objective function. Regions with higher scores are selected for the next round of vertical iterative realignment. Finally, all regions are merged to produce the final realignment result.

Specifically, ReAlign-P scans all columns of the initial alignment, labeling columns composed entirely of residues (without gaps) as 1 and all others as 0. The positions of the first and last “1” are used as cutting points, dividing the alignment vertically into three segments: the head, the region to be realigned, and the tail. Only the targeted segment is subjected to vertical iterative realignment. In contrast, the head and tail regions are retained as-is to preserve potentially meaningful structural features. This strategy minimizes the impact of unstable terminal regions and helps preserve the integrity of the more conserved central region during the realignment process. Following realignment, the corrected targeted segment is concatenated with the unmodified head and tail segments to produce the final alignment. The overall process of ReAlign-P is illustrated in the flowchart (Fig. 1A).

### 2.2 Vertical iterative realignment

In ReAlign-P, the initial input for vertical iterative realignment is the target region extracted from the middle of the alignment. The overall process of vertical iteration is illustrated in Fig. 1B, with the initial input segment labeled as $A_{\mathrm{initial}}$. First, remove all gaps from $A_{\mathrm{initial}}$ to obtain a gap-free sequence set, which is then input into the MSA tool for realignment to produce $A_{\mathrm{realigned}}$. Next, compare $A_{\mathrm{initial}}$ and $A_{\mathrm{realigned}}$ to identify the sub-regions that have either changed or remained the same (unchanged). The unchanged regions are fixed and excluded from further realignment, while the changed sub-regions are used as input for the next iteration.

In each round, ReAlign-P evaluates the objective function scores of the changed sub-region before and after realignment and selects the version with the higher score as the new baseline for the next iteration. The vertical iteration process continues to refine and converge until the current input matches the realignment result ($A_{\mathrm{initial}}=A_{\mathrm{realigned}}$), at which point the iteration terminates. The pseudocode for vertical iterative realignment is shown in Algorithm 1.

Algorithm 1 Vertical iterative realignment

Input: the initial alignment $A_{\mathrm{initial}}\;$will be realigned

Output: the optimized alignment after realignment $A_{\mathrm{final}}$

Function VERTICAL_ITERATIVE_REALIGNMENT($A_{\mathrm{initial}})\;$

1. Preprocessing and realignment:

 ● Remove all gap characters from $A_{\mathrm{initial}}$ to obtain a gap-free sequence set S.

 ● Align S with an MSA tool to obtain $A_{\mathrm{realigned}}$.

2. Termination condition judgment:

 ● If $A_{\mathrm{realigned}}$ is identical to $A_{\mathrm{initial}}$:

   ◯ Return $A_{\mathrm{final}}$

3. Changed and unchanged region detection:

 ● Compare $A_{\mathrm{initial}}$ and $A_{\mathrm{realigned}}$ column-wise to identify regions that are either unchanged or changed.

 ● Let R denote the set of all such regions.

4. Iterative realignment on regions:

   For each region r∈R:

 ● If r is unchanged:

   ◯ Append the corresponding fragment of r from $A_{\mathrm{initial}}$ directly to $A_{\mathrm{final}}$.

 ● Else://r is a changed region

   ◯ Extract the corresponding fragments of region r from $A_{\mathrm{initial}}$ and $A_{\mathrm{realigned}}$.

   ◯ Compute the objective function scores:

     ▪ ${\mathrm{Score}}_{\mathrm{initial}}$ ← objective score of r in $A_{\mathrm{initial}}$

     ▪ ${\mathrm{Score}}_{\mathrm{realigned}}$ ← objective score of r in $A_{\mathrm{realigned}}$

   ◯ If ${\mathrm{Score}}_{\mathrm{initial}}\geq {\mathrm{Score}}_{\mathrm{realigned}}$:

     ▪ ${\mathrm{realigned}}_{r}$ ← VERTICAL_ITERATIVE_REALIGNMENT(fragment of r in $A_{\mathrm{initial}}$)

   ◯ Else:

     ▪ ${\mathrm{realigned}}_{r}$ ← VERTICAL_ITERATIVE_REALIGNMENT(fragment of r in $A_{\mathrm{realigned}}$)

   ◯ Append ${\mathrm{realigned}}_{r}$ to $A_{\mathrm{final}}$

5. Return $A_{\mathrm{final}}$

end Function

**Figure 2.**
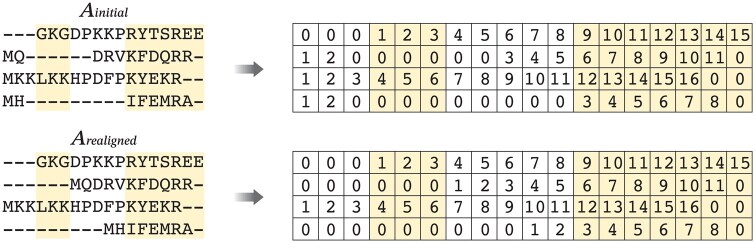
An example illustrating a schematic diagram that identifies the changed and unchanged regions between $A_{\mathrm{initial}}$ and $A_{\mathrm{realigned}}$.

Since the sequence order in $A_{\mathrm{realigned}}$ (produced by the MSA tool) may differ from that in $A_{\mathrm{initial}}$, we first reorder $A_{\mathrm{realigned}}$ to match the sequence order of $A_{\mathrm{initial}}$. This ensures consistency between the two alignments before identifying changed and unchanged regions. To facilitate this detection, we map each alignment fragment from both $A_{\mathrm{initial}}$ and the reordered $A_{\mathrm{realigned}}$ using the following rules: all gaps are labeled as 0, while amino acids are numbered sequentially from left to right based on their position in the original ungapped sequence (i.e. the first amino acid is labeled 1, the second 2, and so on). An example of this mapping is shown in Fig. 2.

After generating the two mapped numerical matrices, we perform a column-wise comparison to identify all column pairs that are perfectly identical across all sequences (highlighted in color in Fig. 2). From these, we extract contiguous column intervals—where the column indices increase consecutively and the interval length is at least 2—as stable, unchanged regions shared by both alignments. Columns that fall outside these intervals are considered changed regions. This approach enables precise localization of conserved and modified areas in the realignment, providing a solid foundation for subsequent comparative analysis and performance evaluation.

### 2.3 Objective function optimization in realignment

During the vertical iterative realignment process, ReAlign-P employs the sum-of-pair (SP) score (Zhai *et al.* 2024b) as the objective function to measure the alignment accuracy of the changed regions in $A_{\mathrm{initial}}$ and $A_{\mathrm{realigned}}$. Assuming that a given change region contains M alignment fragments, each N characters long, ReAlign-P first calculates the score f(l) for the l-th column. The calculation formula is shown in Formula 1.


#(1) f(l)=∑iM∑j≠iMscore(i, j)

In this formula, score(i, j) represents the alignment score between the characters in the i-th and j-th rows. If both characters are amino acids, the score is determined based on the amino acid scoring matrix (Henikoff and Henikoff 1992; Wilbur 1985) provided by the user. If one character is an amino acid and the other is a gap, a penalty is applied according to the user-defined parameter ${\mathrm{score}}_{\mathrm{aa}-\mathrm{gap}}$; if both characters are gaps, a penalty is applied based on the parameter ${\mathrm{score}}_{\mathrm{gap}-\mathrm{gap}}$.

After calculating the score for each column, ReAlign-P sums the scores of all N columns to compute the total SP score for the region. The final SP score calculation formula is shown in Formula 2.


#(2) SP=∑l=0Nf(l)

### 2.4 Implementation and experimental environment

ReAlign-P supports input and output in FASTA format and is implemented in C++17. It integrates two optional MSA tools, MUSCLE5 (Edgar 2022) and FFT-NS-I, into the realignment process. The software was evaluated on an Ubuntu Linux system running on a server with a 2.7 GHz Intel^®^ Xeon^®^ Platinum 8168 CPU and 1 TB of RAM. ReAlign-P is available as an open-source project under the MIT license and can be accessed at https://github.com/malabz/ReAlign-P. The DOI of Figshare is 10.6084/m9.figshare.29432357.

### 2.5 The benchmark to evaluate ReAlign-P

To evaluate the performance of ReAlign-P, this study selected four widely used benchmark protein datasets: BAliBASE v3 (BAliBASE) (Thompson *et al.* 2005), OXBench (Raghava *et al.* 2003), PREFAB4, and SABRE. BAliBASE v3 includes 386 test cases based on 3D structural superposition. It offers high-quality reference alignments that are manually refined and cover a wide range of representative protein fold types. OXBench contains 395 structure-based alignment cases, suitable for evaluating structurally conserved region (SCR) recognition, domain alignment, and the overall accuracy of MSA tools. PREFAB4 includes 1682 alignment cases built from consensus structural alignments produced by FSSP and CE and can be expanded by adding up to 48 homologous sequences retrieved through database searches. SABRE, derived from SABmark v1.65 (Van Walle *et al.* 2005), consists of 423 test cases and is particularly well-suited for assessing alignment accuracy among distantly related homologous proteins. Its reference alignments are generated using two structural alignment algorithms: SOFI and CE. The details of the four datasets are summarized in Table 1, available as supplementary data at *Bioinformatics* online. “Alignments” indicates the number of alignment tasks in each dataset. “Average sequences” refers to the average number of sequences per alignment, calculated by dividing the total number of sequences across all alignments by the number of alignments. “Average length” represents the average sequence length, computed by first determining the average sequence length within each alignment and then taking the average of these values.

All four datasets are publicly available at http://www.drive5.com/bench/. In each dataset, test alignment files are located in the in folder, and reference alignment files are in the ref folder. The evaluation metrics include the Q score (the proportion of correctly aligned base pairs relative to those in the reference) and the TC score (the proportion of correctly aligned columns relative to those in the reference). The base case is ignored during the calculation.

## 3 Results

### 3.1 MUSCLE5 as the default MSA tool in vertical iterative realignment

We evaluated two tools, FFT-NS-I and MUSCLE5, to assist users in selecting the one that most effectively improves alignment quality during vertical iterations. The objective function used the BLOSUM62 and PAM250 protein scoring matrices. The amino acid–gap penalty (${\mathrm{score}}_{\mathrm{aa}-\mathrm{gap}}$) ranged from 0 to −10, specifically taking values of 0, −1, −2, −3, −4, −5, −6, −7, −8, −9, and −10. The gap–gap penalty (${\mathrm{score}}_{\mathrm{gap}-\mathrm{gap}}$) was fixed at 0. This ensures the broad applicability of the MSA tool in vertical iterative realignment. All parameter combinations were tested in this study. The test datasets generated initial alignments for four benchmark datasets: BAliBASE, OXBench, SABRE, and PREFAB4. These alignments were generated using ten different parameter configurations (Table 2, available as supplementary data at *Bioinformatics* online) across six MSA tools: Clustal Omega (Sievers and Higgins 2014), FAMSA, MAFFT, MUSCLE, ProbCons, and T-Coffee (Notredame *et al.* 2000). The initial alignments were then fed into ReAlign-P and tested with all parameter combinations. Finally, the accuracy of the benchmark datasets under all parameter combinations was evaluated using the average Q and TC scores.

**Figure 3.**
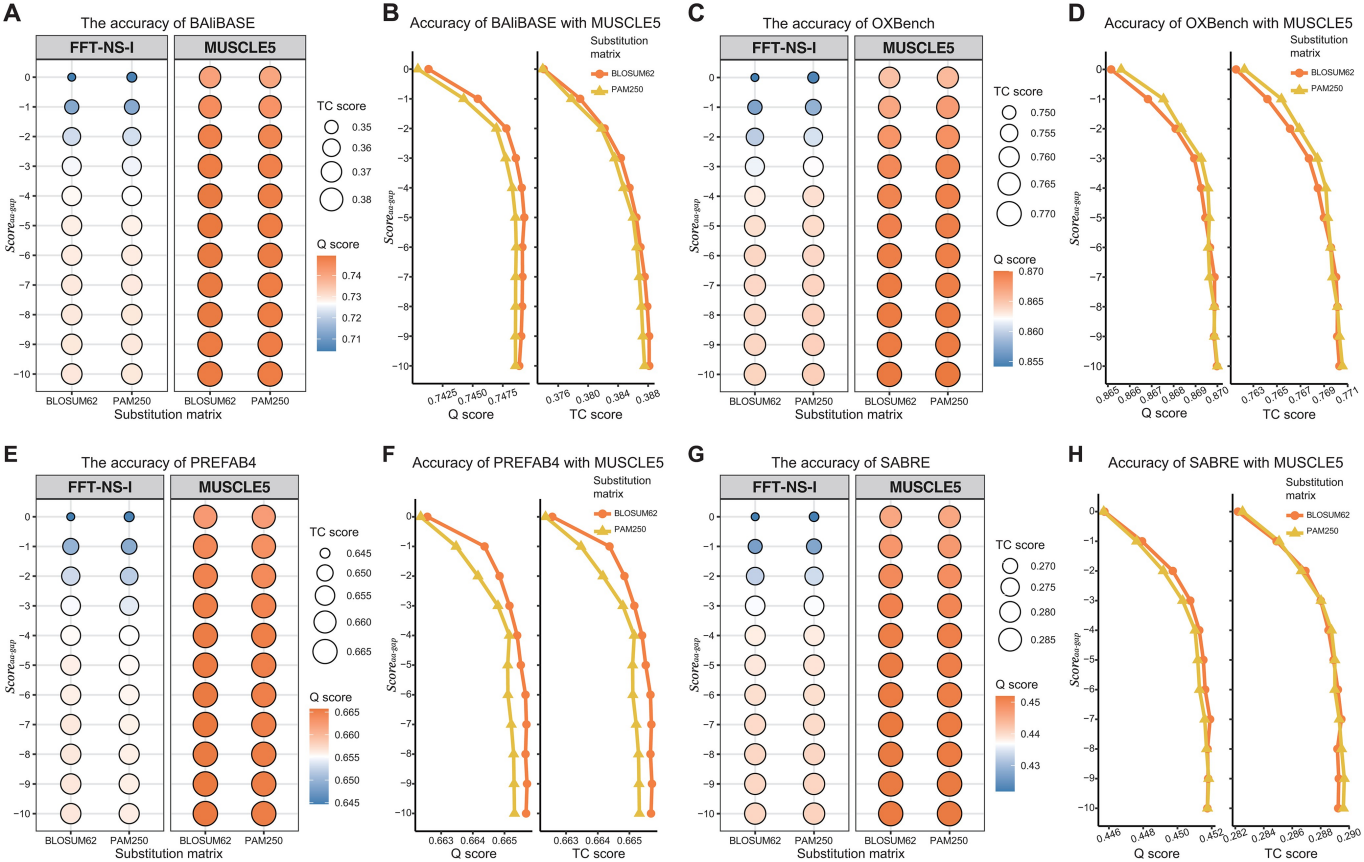
Accuracy of various parameters across four benchmark datasets. On the BAliBASE dataset, vertical iterative realignment was performed using FFT-NS-I and MUSCLE5 as the base aligners. Q and TC scores were evaluated under 11 penalty scores and two protein scoring matrices (A). Similarly, using MUSCLE5 as the aligner, Q and TC scores were calculated under the same 11 penalty scores and two matrices (B). Corresponding analysis of OXBench (C, D), PREFAB4 (E, F), and SABRE (G, H) datasets. The BAliBASE, OXBench, PREFAB4, and SABRE datasets contain 386, 395, 1682, and 423 test alignments, respectively. The heat bubble chart displays the average Q and TC scores across all test alignments in each dataset, based on realignments of the initial results produced by 10 different MSA tools under various parameter combinations. For instance, in the BAliBASE dataset, each bubble represents the average results from 386 test alignments aligned using the 10 MSA tools. In the chart, bubble color indicates the Q score, while bubble size corresponds to the TC score. The points on the line graph also represent the mean values of all initial alignments.

For each benchmark dataset, we calculated the average Q and TC scores of alignments optimized from all initial alignments across different parameter combinations. The results show that for all benchmark datasets, the average Q and TC scores are higher when MUSCLE5 is used as the MSA tool in vertical iterative realignment compared to FFT-NS-I (Fig. 3A, C, E, G). This indicates that MUSCLE5 is more effective in improving alignment quality. As a result, MUSCLE5 is selected as the default MSA tool in the vertical iterative realignment stage of ReAlign-P to ensure optimal performance.

### 3.2 Selection and configuration of default parameters for the objective function in vertical iterative realignment

After designating MUSCLE5 as the default MSA tool for the vertical iterative realignment stage, we proceeded to determine the default parameters for the objective function based on the experiments outlined above, with particular attention to the protein scoring matrix and the ${\mathrm{score}}_{\mathrm{aa}-\mathrm{gap}}$ penalty value. We offered two well-established protein scoring matrices, BLOSUM62 and PAM250, both of which have demonstrated strong performance in a wide range of bioinformatics applications. For the amino acid–gap penalty (${\mathrm{score}}_{\mathrm{aa}-\mathrm{gap}}$), we conducted a grid search over values ranging from 0 to −10. The gap–gap penalty (${\mathrm{score}}_{\mathrm{gap}-\mathrm{gap}}$) was fixed at 0. These steps enabled us to identify the optimal values for most datasets.

We evaluated 22 combinations of protein scoring matrices and ${\mathrm{score}}_{\mathrm{aa}-\mathrm{gap}}$ penalties, calculating the mean Q and TC scores for all optimized initial alignments across each benchmark dataset. For the BAliBASE and PREFAB4 datasets, the Q and TC scores improved significantly as ${\mathrm{score}}_{\mathrm{aa}-\mathrm{gap}}$ decreased. When ${\mathrm{score}}_{\mathrm{aa}-\mathrm{gap}}$ reached −6 or lower, the scores stabilized. Notably, when BLOSUM62 was assessed for amino acid scoring, the Q and TC scores consistently outperformed those obtained with PAM250, regardless of the ${\mathrm{score}}_{\mathrm{aa}-\mathrm{gap}}$ value (Fig. 3B, F). On the OXBench dataset, the Q and TC scores also improved as ${\mathrm{score}}_{\mathrm{aa}-\mathrm{gap}}$ decreased. Again, once ${\mathrm{score}}_{\mathrm{aa}-\mathrm{gap}}$ reached −6 or lower, the scores stabilized. At this point, the accuracy with BLOSUM62 and PAM250 was nearly identical (Fig. 3D). For the SABRE dataset, the Q and TC scores increased gradually with decreasing ${\mathrm{score}}_{\mathrm{aa}-\mathrm{gap}}$, peaking at −7, where the highest Q scores were achieved with BLOSUM62 as the amino acid scoring matrix (Fig. 3H).

Considering all the datasets, the combination of BLOSUM62 as the protein scoring matrix and ${\mathrm{score}}_{\mathrm{aa}-\mathrm{gap}}$ set to −6 yielded high accuracy across the majority of benchmark datasets. As a result, we selected this combination as the default parameter for the ReAlign-P objective function.

### 3.3 Improvement of initial alignment accuracy

ReAlign-P is a realignment tool developed to enhance the accuracy of initial MSA. To assess its effectiveness, we used the same initial alignments as in the previous experiment. Among them, the alignments generated by ClustalO, FAMSA1, FFT-NS-2, ProbCons1, and T-Coffee skip their respective iterative optimization phases, while those generated by FAMSA2, FFT-NS-I, MUSCLE3, MUSCLE5, and ProbCons2 involve iterative optimization during the alignment process. In this experiment, ReAlign-P was applied with its default settings: MUSCLE5 was used as the MSA tool during the vertical iterative realignment stage, BLOSUM62 served as the amino acid scoring matrix, the amino acid–gap penalty (${\mathrm{score}}_{\mathrm{aa}-\mathrm{gap}}$) was set to −6, and the gap–gap penalty (${\mathrm{score}}_{\mathrm{gap}-\mathrm{gap}}$) was set to 0 in the objective function. We evaluated performance on four benchmark datasets, calculating the average Q and TC scores for all test cases before and after realignment.

**Figure 4.**
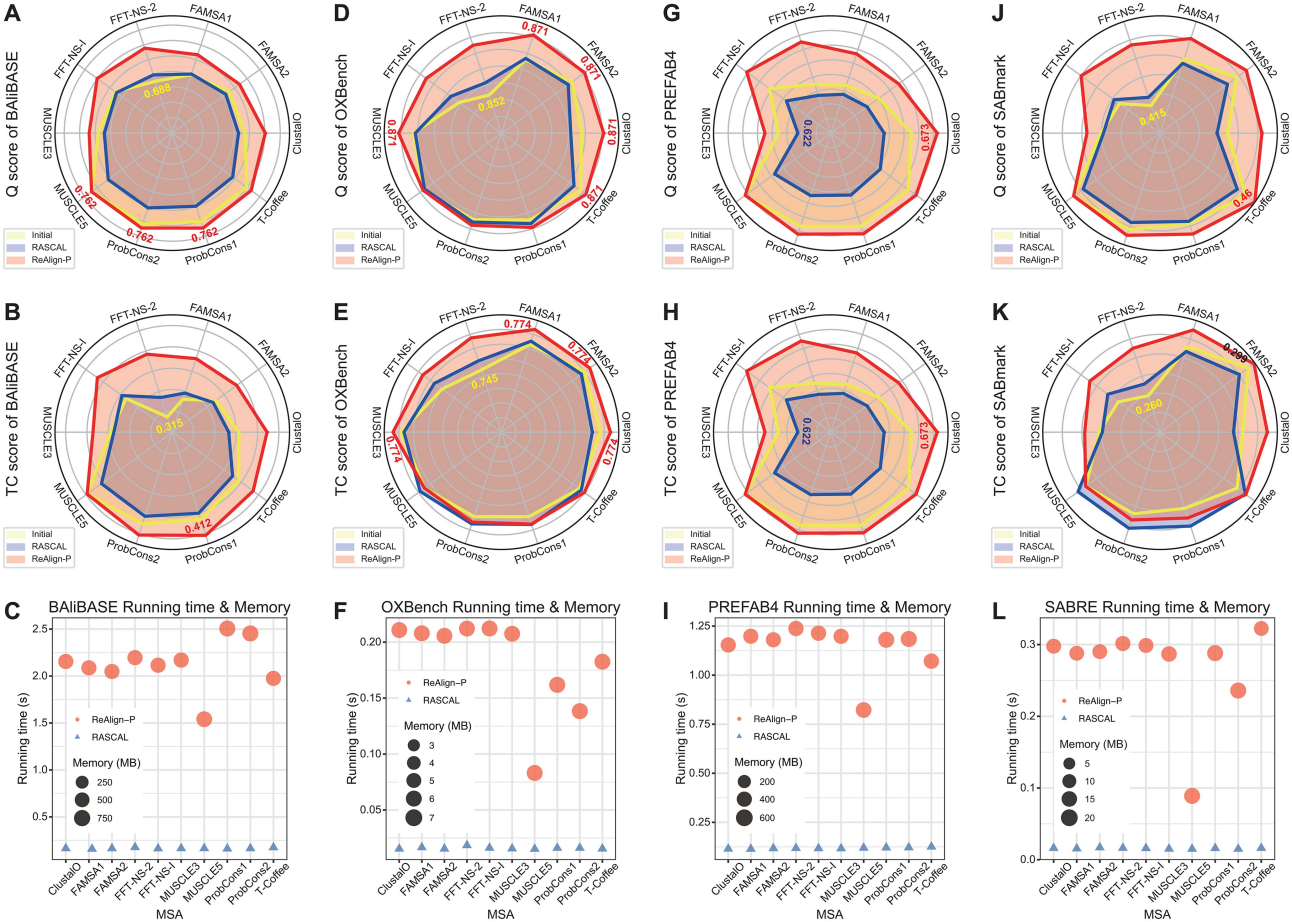
Performance comparison based on four benchmark datasets. Comparison of Q (A) and TC scores (B) for initial alignments from 10 MSA tools, realignments by ReAlign-P, and realignments by RASCAL on the BAliBASE dataset. C. Comparison of running time and memory usage between ReAlign-P and RASCAL on the BAliBASE dataset. Corresponding analysis of OXBench (D–F), PREFAB4 (G–I), and SABRE (J–L) datasets. The BAliBASE, OXBench, PREFAB4, and SABRE datasets contain 386, 395, 1682, and 423 test alignments, respectively. The Q and TC scores shown in the radar charts represent the averages across all test alignments in each dataset. The values shown in the figures represent the maximum and minimum among these average values. If there are multiple identical maximum or minimum values, they are also indicated in the figures. The bubble charts illustrate the average running time and memory usage for all test alignments in each dataset when ReAlign-P and RASCAL realign the initial alignments generated by the 10 MSA tools. In these charts, the bubble height indicates running time, while the bubble size reflects memory consumption.

On the BAliBASE and PREFAB4 datasets, ReAlign-P consistently improved Q (Fig. 4A, G, Table 3, available as supplementary data at *Bioinformatics* online) and TC (Fig. 4B, H, Table 4, available as supplementary data at *Bioinformatics* online) scores after realignment, regardless of whether the initial alignments were generated with or without internal iterative optimization. For the OXBench dataset, ReAlign-P significantly increased the Q score, particularly for alignments produced by ClustalO, FFT-NS-I, and FFT-NS-2 (Fig. 4D, Table 3, available as supplementary data at *Bioinformatics* online). While the TC score also improved, the gains were more modest (Fig. 4E, Table 4, available as supplementary data at *Bioinformatics* online). In the SABRE dataset, ReAlign-P notably enhanced both Q and TC scores, with the most substantial improvements observed in initial alignments from ClustalO, FFT-NS-I, and FFT-NS-2 (Fig. 4J, K, Tables 3 and 4, available as supplementary data at *Bioinformatics* online). These results demonstrate that ReAlign-P can effectively boost alignment accuracy, regardless of whether the initial MSA was generated with iterative refinement. It also helps close the performance gap between different configurations of the same MSA tool, compensating for the difference in internal iterative optimization.

### 3.4 ReAlign-P outperforms RASCAL in alignment quality

In this experiment, we used initial alignments generated under 10 different parameter configurations across all benchmark datasets. These alignments were then processed by RASCAL and ReAlign-P for realignment. RASCAL was run with its default parameters (Table 2, available as supplementary data at *Bioinformatics* online), while ReAlign-P used the default configuration determined in the previous experiment: MUSCLE5 as the MSA tool during the vertical iteration realignment phase, BLOSUM62 as the protein scoring matrix, the amino acid–gap penalty (${\mathrm{score}}_{\mathrm{aa}-\mathrm{gap}}$) was set to −6, and the gap–gap penalty (${\mathrm{score}}_{\mathrm{gap}-\mathrm{gap}}$) was set to 0 in the objective function. To assess the alignment optimization performance of the two tools, we calculated the average Q and TC scores across all test cases for each dataset after realignment. Additionally, to provide a comprehensive performance comparison, we recorded both running time and memory usage using the system command/usr/bin/time −v, where “Elapsed (wall clock) time” reflects the actual execution time, and “Maximum resident set size” indicates peak memory consumption.

On the BAliBASE and PREFAB4 datasets, ReAlign-P achieved significantly higher Q and TC scores after realignment compared to RASCAL (Fig. 4A, B, G, H, Tables 3 and 4, available as supplementary data at *Bioinformatics* online). Notably, on the BAliBASE dataset, RASCAL’s optimization of initial alignments produced by ClustalO, FAMSA2, MUSCLE3, MUSCLE5, ProbCons1, ProbCons2, and T-Coffee led to lower Q and TC scores than the original unoptimized alignments (Fig. 4A, B, Tables 3 and 4, available as supplementary data at *Bioinformatics* online). A similar trend was observed on the PREFAB4 dataset, where RASCAL’s optimized alignments consistently underperformed the initial ones in both Q and TC scores, indicating suboptimal results (Fig. 4G, H, Tables 3 and 4, available as supplementary data at *Bioinformatics* online).

For the OXBench dataset, ReAlign-P consistently delivered higher Q scores than RASCAL across all initial alignments (Fig. 4D, Table 3, available as supplementary data at *Bioinformatics* online). In terms of TC scores, RASCAL slightly outperformed ReAlign-P only when optimizing alignments generated by MUSCLE5 or ProbCons2; in all other cases, ReAlign-P’s TC scores were either higher or on par (Fig. 4E, Table 4, available as supplementary data at *Bioinformatics* online). Additionally, RASCAL’s optimization of ClustalO alignments resulted in declines in both Q and TC scores. On the SABRE dataset, ReAlign-P again achieved higher Q scores across all initial alignments generated by the ten tools (Fig. 4J, Table 3, available as supplementary data at *Bioinformatics* online). For TC scores, RASCAL showed slight advantages only on alignments from MUSCLE5, ProbCons1, and ProbCons2, while ReAlign-P outperformed or matched RASCAL in all other cases (Fig. 4K, Table 4, available as supplementary data at *Bioinformatics* online). Moreover, RASCAL's optimization led to decreases in both Q and TC scores for alignments from ClustalO, FAMSA1, FAMSA2, and MUSCLE3, and caused drops in Q scores for MUSCLE5, ProbCons1, ProbCons2, and T-Coffee.

In terms of running time performance, ReAlign-P has slightly higher time and memory requirements than RASCAL, although both are within an acceptable range (Fig. 4C, F, I, L). More importantly, ReAlign-P consistently delivers greater improvements in alignment accuracy, particularly in Q scores, which remain stable and reliable. Unlike RASCAL, which occasionally suffers from “negative optimization” that degrades alignment quality, ReAlign-P maintains a consistent and robust positive optimization effect.

## 4 Discussion

Most existing protein realignment tools are outdated or unsupported. This study presents ReAlign-P, a robust post-processing tool using vertical iterative realignment. By dividing alignments into head, middle, and tail regions, focuses on optimizing the middle section to enhance accuracy. ReAlign-P’s performance was systematically evaluated on four benchmark datasets: BAliBASE, OXBench, PREFAB, and SABmark.

We adopted a step-by-step strategy to determine the default MSA tool and objective function parameters for ReAlign-P's vertical iterative realignment. First, we selected the basic MSA tool, as its alignment quality directly influences the effectiveness of subsequent optimization. Next, we determined the appropriate objective function configuration based on the chosen MSA tool, including the protein scoring matrix and corresponding penalty parameters. Notably, We focused solely on testing the penalty between amino acids and gaps, while the alignment score for gap-gap pairs was always set to 0. This setting was designed to preserve the integrity of the gap structure in the sequence and prevent the unnecessary influence of gap-gap pairings. Through this approach, we ensure that ReAlign-P maintains high robustness across different datasets.

We applied ReAlign-P and RASCAL (the only available alternative) with default settings to optimize alignments across four datasets. ReAlign-P consistently improved or maintained Q and TC scores, showing stable performance, while RASCAL often reduced accuracy, indicating limited robustness. Although ReAlign-P uses MUSCLE5 and iterative optimization—resulting in slightly higher resource use—the trade-off is justified by its superior alignment quality and reliability.

## Supplementary Material

btaf421_Supplementary_Data

## References

[btaf421-B1] Anson EL , Myers EW. ReAligner: A program for refining DNA sequence multi-alignments. J Comput Biol 1997;4:369–83. 10.1089/cmb.1997.4.369 9278066

[btaf421-B2] Brudno M , Do CB, Cooper GM, NISC Comparative Sequencing Program et al LAGAN and Multi-LAGAN: efficient tools for large-scale multiple alignment of genomic DNA. Genome Res 2003;13:721–31. 12654723 10.1101/gr.926603 PMC430158

[btaf421-B3] Chakrabarti S , Lanczycki CJ, Panchenko AR et al Refining multiple sequence alignments with conserved core regions. Nucleic Acids Res 2006;34:2598–606. 16707662 10.1093/nar/gkl274 PMC1463900

[btaf421-B4] Chao J , Tang F, Xu L et al Developments in algorithms for sequence alignment: a review. Biomolecules 2022;12:546. 35454135 10.3390/biom12040546 PMC9024764

[btaf421-B5] Deorowicz S , Debudaj-Grabysz A, Gudyś A et al FAMSA: fast and accurate multiple sequence alignment of huge protein families. Sci Rep 2016;6:33964. 27670777 10.1038/srep33964 PMC5037421

[btaf421-B6] Do CB , Mahabhashyam MSP, Brudno M et al ProbCons: probabilistic consistency-based multiple sequence alignment. Genome Res 2005;15:330–40. 15687296 10.1101/gr.2821705 PMC546535

[btaf421-B7] Edgar RC. MUSCLE: multiple sequence alignment with high accuracy and high throughput. Nucleic Acids Res 2004;32:1792–7. 15034147 10.1093/nar/gkh340 PMC390337

[btaf421-B8] Edgar RC. Muscle5: high-accuracy alignment ensembles enable unbiased assessments of sequence homology and phylogeny. Nat Commun 2022;13:6968. 36379955 10.1038/s41467-022-34630-w PMC9664440

[btaf421-B9] Gudyś A , Deorowicz S. QuickProbs 2: towards rapid construction of high-quality alignments of large protein families. Sci Rep 2017;7:41553. 28139687 10.1038/srep41553 PMC5282490

[btaf421-B10] Henikoff S , Henikoff JG. Amino acid substitution matrices from protein blocks. Proc Natl Acad Sci U S A 1992;89:10915–9. 1438297 10.1073/pnas.89.22.10915 PMC50453

[btaf421-B11] Katoh K , Standley DM. MAFFT multiple sequence alignment software version 7: improvements in performance and usability. Mol Biol Evol 2013;30:772–80. 23329690 10.1093/molbev/mst010 PMC3603318

[btaf421-B12] Liu Y , Schmidt B, Maskell DL et al MSAProbs: multiple sequence alignment based on pair hidden markov models and partition function posterior probabilities. Bioinformatics 2010;26:1958–64. 20576627 10.1093/bioinformatics/btq338

[btaf421-B13] Lyras DP , Metzler D. ReformAlign: improved multiple sequence alignments using a profile-based meta-alignment approach. BMC Bioinformatics 2014;15:265–18. 25099134 10.1186/1471-2105-15-265 PMC4133627

[btaf421-B14] Manohar A, Batzoglou S 2005. TreeRefiner: a tool for refining a multiple alignment on a phylogenetic tree. In: *2005 IEEE Computational Systems Bioinformatics Conference (CSB'05)*. IEEE. 111–19. 10.1109/csb.2005.53 16447969

[btaf421-B15] Mokaddem A , Bel Hadj A, Elloumi M et al Refin-Align: new refinement algorithm for multiple sequence alignment. Informatica 2019;43:527–34.

[btaf421-B16] Notredame C , Higgins DG, Heringa J et al T-Coffee: a novel method for fast and accurate multiple sequence alignment. J Mol Biol 2000;302:205–17. 10964570 10.1006/jmbi.2000.4042

[btaf421-B17] Raghava GPS , Searle SMJ, Audley PC et al OXBench: a benchmark for evaluation of protein multiple sequence alignment accuracy. BMC Bioinform 2003;4:47–23. 10.1186/1471-2105-4-47 PMC280650 14552658

[btaf421-B18] Sievers F , Higgins DG. Clustal omega. Curr Protoc Bioinform 2014;48:13.1–13.16. 3 10.1002/0471250953.bi0313s48 25501942

[btaf421-B19] Thompson JD , Koehl P, Ripp R et al BAliBASE 3.0: latest developments of the multiple sequence alignment benchmark. Proteins 2005;61:127–36. 16044462 10.1002/prot.20527

[btaf421-B20] Thompson JD , Thierry JC, Poch O et al RASCAL: rapid scanning and correction of multiple sequence alignments. Bioinformatics 2003;19:1155–61. 12801878 10.1093/bioinformatics/btg133

[btaf421-B21] Van Walle I , Lasters I, Wyns L et al SABmark—a benchmark for sequence alignment that covers the entire known fold space. Bioinformatics 2005;21:1267–8. 15333456 10.1093/bioinformatics/bth493

[btaf421-B22] Wallace IM , O’Sullivan O, Higgins DG et al Evaluation of iterative alignment algorithms for multiple alignment. Bioinformatics 2005;21:1408–14. 15564300 10.1093/bioinformatics/bti159

[btaf421-B23] Wang Y , Zhai Y, Ding Y et al SBSM-Pro: support bio-sequence machine for proteins. Sci China Inf Sci 2024;67:212106.

[btaf421-B24] Wei Q , Zou H, Zhong C et al RPfam: a refiner towards curated-like multiple sequence alignments of the pfam protein families. J Bioinform Comput Biol 2022;20:2240002. 35430947 10.1142/S0219720022400029

[btaf421-B25] Wicker N , Perrin GR, Thierry JC et al Secator: a program for inferring protein subfamilies from phylogenetic trees. Mol Biol Evol 2001;18:1435–41. 11470834 10.1093/oxfordjournals.molbev.a003929

[btaf421-B26] Wilbur WJ. On the PAM matrix model of protein evolution. Mol Biol Evol 1985;2:434–47. 3870870 10.1093/oxfordjournals.molbev.a040360

[btaf421-B27] Zhai Y , Chao J, Wang Y et al TPMA: a two pointers meta-alignment tool to ensemble different multiple nucleic acid sequence alignments. PLOS Comput Biol 2024b;20:e1011988. 38557416 10.1371/journal.pcbi.1011988 PMC11008887

[btaf421-B28] Zhai Y , Zhou T, Wei Y et al ReAlign-N: an integrated realignment approach for multiple nucleic acid sequence alignment, combining global and local realignments. NAR Genomics Bioinform 2024a;6:lqae170. 10.1093/nargab/lqae170 PMC11655299 39703429

[btaf421-B29] Zhan Q , Fu Y, Jiang Q et al SpliVert: a protein multiple sequence alignment refinement method based on splitting-splicing vertically. Protein Pept Lett 2020;27:295–302. 31385760 10.2174/0929866526666190806143959

[btaf421-B30] Zhang Z, Lin H, Li M. 2007. “MANGO: a new approach to multiple sequence alignment,” Comput Syst Bioinform: 6:237–47. 17951828

[btaf421-B31] Zhou T , Zhang P, Zou Q et al HAlign 4: a new strategy for rapidly aligning millions of sequences. Bioinformatics 2024;40:btae718. 39607773 10.1093/bioinformatics/btae718 PMC11646084

