# Supplementary material for: ReAlign-P: a vertical iterative realignment method for protein multiple sequence alignment
Source: Bioinformatics. 2025 Jul 25;41(8):btaf421. doi: 10.1093/bioinformatics/btaf421 (PMC12342757; doi:10.1093/bioinformatics/btaf421)
Supplement: btaf421_Supplementary_Data [file btaf421_supplementary_data.docx]

**Supplementary material for**

**ReAlign-P: A vertical iterative realignment method for protein multiple sequence alignment**

Yixiao Zhai^1,2^, Pinglu Zhang^1,2^, Quan Zou^1,2^, Ximei Luo^1,*^

^1^Institute of Fundamental and Frontier Sciences, University of Electronic Science and Technology of China, Chengdu, 610054, China

^2^Yangtze Delta Region Institute (Quzhou), University of Electronic Science and Technology of China, Quzhou, 324003, China

*Corresponding author E-mail: luoximei@uestc.edu.cn.

This file includes:

Table S1, S2, S3 and S4.

Table S1. The summary of datasets

| Dataset | Alignments | Average sequences | Average length |
| --- | --- | --- | --- |
| BAliBASE | 386 | 28.71 | 338.28 |
| OXBench | 395 | 8.33 | 138.58 |
| PREFAB4 | 1682 | 45.19 | 233.51 |
| SABRE | 423 | 5.72 | 171.22 |

Table S2. Detailed software versions and execution commands

| Software versions | Tool | Running command |
| --- | --- | --- |
| ClustalO v1.2.4 | ClustalO | clustalo -i test.fa -o test_clustalo.fa |
| FAMSA v2.2.3 | FAMSA1 | famsa -refine_mode off test.fa > test_famsa1.fa |
|  | FAMSA2 | famsa -refine_mode on test.fa > test_famsa2.fa |
| MAFFT v7.526 | FFT-NS-2 | mafft --retree 2 test.fa > test_fftns2.fa |
|  | FFT-NS-I | fftnsi test.fa > test_fftnsi.fa |
| MUSCLE v3.8.1551 | MUSCLE3 | muscle -in test.fa -out test_muscle3.fa |
| MUSCLE v5.3 | MUSCLE5 | muscle -super5 test.fa -output test_muscle5.fa |
| ProbCons v1.12 | ProbCons1 | probcons -ir 0 test.fa > test_probcons1.fa |
|  | ProbCons2 | probcons test.fa > test_probcons2.fa |
| T-Coffee v13.46 | T-Coffee | t_coffee -infile test.fa -outfile test_tcoffee.fa -output fasta |
| RASCAL v1.34 | RASCAL | rascal test_msa.fa test_msa_rascal.fa |

Table S3. The average Q score of the benchmark datasets.

| Tool | BAliBASE | | | OXBench | | | PREFAB4 | | | SABRE | | |
| --- | --- | --- | --- | --- | --- | --- | --- | --- | --- | --- | --- | --- |
|  | Initial | RASCAL | ReAlign-P | Initial | RASCAL | ReAlign-P | Initial | RASCAL | ReAlign-P | Initial | RASCAL | ReAlign-P |
| ClustalO | 0.718 | 0.707 | **0.751** | 0.864 | 0.861 | **0.871** | 0.655 | 0.636 | **0.673** | 0.435 | 0.429 | **0.452** |
| FAMSA1 | 0.699 | 0.701 | **0.733** | 0.863 | 0.863 | **0.871** | 0.634 | 0.628 | **0.657** | 0.440 | 0.438 | **0.451** |
| FAMSA2 | 0.714 | 0.707 | **0.735** | 0.866 | 0.865 | **0.871** | 0.640 | 0.631 | **0.657** | 0.449 | 0.443 | **0.455** |
| FFT-NS-2 | 0.688 | 0.699 | **0.745** | 0.852 | 0.856 | **0.868** | 0.635 | 0.627 | **0.665** | 0.415 | 0.419 | **0.447** |
| FFT-NS-I | 0.714 | 0.712 | **0.750** | 0.856 | 0.859 | **0.868** | 0.652 | 0.637 | **0.671** | 0.426 | 0.429 | **0.450** |
| MUSCLE3 | 0.720 | 0.710 | **0.735** | 0.866 | 0.866 | **0.871** | 0.636 | 0.622 | **0.645** | 0.430 | 0.429 | **0.437** |
| MUSCLE5 | 0.761 | 0.728 | **0.762** | **0.869** | 0.868 | **0.869** | **0.672** | 0.647 | **0.672** | **0.455** | 0.449 | **0.455** |
| ProbCons1 | 0.750 | 0.725 | **0.762** | 0.867 | 0.868 | **0.870** | 0.667 | 0.644 | **0.672** | 0.449 | 0.447 | **0.454** |
| ProbCons2 | 0.754 | 0.727 | **0.762** | 0.867 | 0.868 | **0.869** | 0.667 | 0.645 | **0.672** | 0.452 | 0.448 | **0.455** |
| T-Coffee | 0.749 | 0.721 | **0.758** | 0.869 | 0.867 | **0.871** | 0.665 | 0.642 | **0.672** | 0.454 | 0.449 | **0.460** |

Table S4. The average TC score of the benchmark datasets.

| Tool | BAliBASE | | | OXBench | | | PREFAB4 | | | SABRE | | |
| --- | --- | --- | --- | --- | --- | --- | --- | --- | --- | --- | --- | --- |
|  | Initial | RASCAL | ReAlign-P | Initial | RASCAL | ReAlign-P | Initial | RASCAL | ReAlign-P | Initial | RASCAL | ReAlign-P |
| ClustalO | 0.363 | 0.353 | **0.389** | 0.768 | 0.762 | **0.774** | 0.655 | 0.636 | **0.673** | 0.282 | 0.278 | **0.295** |
| FAMSA1 | 0.332 | 0.339 | **0.372** | 0.763 | 0.765 | **0.774** | 0.634 | 0.628 | **0.657** | 0.286 | 0.283 | **0.295** |
| FAMSA2 | 0.350 | 0.347 | **0.375** | 0.770 | 0.767 | **0.774** | 0.640 | 0.631 | **0.657** | 0.296 | 0.290 | **0.299** |
| FFT-NS-2 | 0.315 | 0.334 | **0.377** | 0.745 | 0.751 | **0.768** | 0.635 | 0.627 | **0.665** | 0.260 | 0.266 | **0.285** |
| FFT-NS-I | 0.354 | 0.358 | **0.387** | 0.750 | 0.757 | **0.766** | 0.652 | 0.637 | **0.671** | 0.266 | 0.273 | **0.284** |
| MUSCLE3 | 0.358 | 0.355 | **0.374** | 0.769 | 0.767 | **0.774** | 0.636 | 0.622 | **0.645** | 0.271 | 0.270 | **0.279** |
| MUSCLE5 | **0.399** | 0.382 | **0.399** | 0.765 | **0.769** | 0.765 | **0.672** | 0.647 | **0.672** | 0.287 | **0.292** | 0.287 |
| ProbCons1 | 0.386 | 0.380 | **0.402** | 0.761 | **0.766** | **0.766** | 0.667 | 0.644 | **0.672** | 0.281 | **0.291** | 0.286 |
| ProbCons2 | 0.390 | 0.383 | **0.401** | 0.761 | **0.766** | 0.765 | 0.667 | 0.645 | **0.672** | 0.284 | **0.292** | 0.287 |
| T-Coffee | 0.377 | 0.370 | **0.394** | 0.766 | 0.767 | **0.770** | 0.665 | 0.642 | **0.672** | 0.289 | **0.294** | **0.294** |
